# The impact of job and family demands on partner’s fatigue: A study of Japanese dual-earner parents

**DOI:** 10.1371/journal.pone.0172291

**Published:** 2017-02-24

**Authors:** Mayumi Watanabe, Akihito Shimazu, Arnold B. Bakker, Evangelia Demerouti, Kyoko Shimada, Norito Kawakami

**Affiliations:** 1 Graduate School of Health Management, Keio University, Fujisawa, Japan; 2 Department of Mental Health, The University of Tokyo, Graduate School of Medicine, Tokyo, Japan; 3 Center of Excellence for Positive Organizational Psychology, Erasmus University Rotterdam, Rotterdam, The Netherlands; 4 Department of Management, University of Johannesburg, Auckland Park, South Africa; 5 Department of Industrial Engineering and Innovation Sciences, Eindhoven University of Technology, Eindhoven, The Netherlands; 6 Faculty of Sociology, Toyo University, Tokyo, Japan; National Center of Neurology and Psychiatry, JAPAN

## Abstract

**Objectives:**

This study of Japanese dual-earner couples examined the impact of family and job demands on one’s own and one’s partner’s fatigue as well as gender differences in these effects.

**Methods:**

A total of 2,502 parents (1,251 couples) were surveyed using a self-administered questionnaire. A crossover model was tested using structural equation modeling.

**Results:**

The results of structural equation modeling analyses showed that both job and family demands independently exacerbated fatigue. There was an indirect effect of job and family demands on partner fatigue through one’s own fatigue only from husbands to wives. An indirect effect of job demands on partner fatigue through partner’s family demands was identified only from wives to husbands. Furthermore, there were gender differences in the crossover of fatigue.

**Conclusions:**

This study shows that job and family demands influence family circumstances. When considering means to reduce employees’ fatigue, gender differences in the mechanism of fatigue need to be taken into account.

## Introduction

In recent decades, there has been a considerable increase in the participation of women in the workforce, leading to a higher prevalence of dual-earner couples. These couples, especially those with children, often have heavy responsibilities in the management of family and work tasks. According to the effort-recovery model[1], the process of expending effort causes load reactions, which, under the condition of sufficient recovery during the nonworking period, stabilize at a baseline level within a short time. Although downtime from work usually performs this recovery function[2], this is often inhibited for dual-earner parents. Insufficient recovery invokes accumulated fatigue, which may lead to health deterioration[3], and impairment in children’s well-being[4].

The fatigue of dual-earner parents may be influenced by not only their own work and family demands but also by those demands on their partner, which is referred to as “crossover”[5–8]. Evidence has accumulated over the past two decades for the existence of crossover of health and well-being between partners in dual-earner couples[9–14]. Similarly, demands on parents may be interdependent. Because parents usually share common stressors, there should be a positive correlation between partners’ family demands. Previous findings provide evidence of common stressors such as economic hardship playing a significant role in the mechanism of the crossover effect[15,16]. Furthermore, job demands on both parents are likely to show a positive relationship with each other. Morrison[17] suggests that, as a consequence of the fact that similar individuals are more likely to marry each other, partners are likely to have jobs of similar status that place comparable demands on each of them.

We wished to add to previous studies on the crossover of dual-earner parents’ health and well-being by testing the crossover of fatigue between partners after accounting for the impact of each partner’s work and family demands. To examine this effect in detail, we analyzed dyads instead of individuals separately. This also allowed us to investigate how these effects differ as a function of gender.

**Fig 1:**
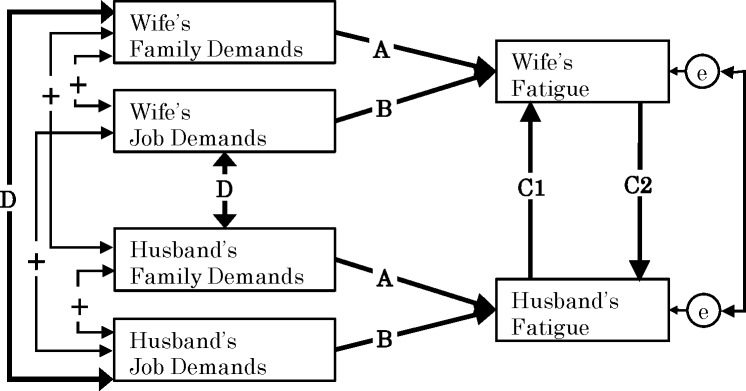
Hypothesis model.

Fig 1 depicts the framework of this study. Please note that we chose fatigue as an indicator of health and well-being because, as explained above, dual-earner parents are likely to face serious challenges in the process of recovering from work-related fatigue.

The effect of job demands on employees’ own fatigue can be explained using the framework of the job demands-resources (JD-R) model. In this model, job demands are defined as ‘‘physical, social, or organizational job aspects that require sustained physical and/or psychological effort and are associated with certain physiological and/or psychological costs”[18]. Job demands have been identified as the main cause of burnout, which in turn leads to poor health[19]. Evidence has confirmed this association[18,20,21]. Family demands have also been identified as a detrimental factor to one’s own health and well-being[22–24]. Consequently, we consider that an employee’s job and family demands will have an undesirable impact on their own fatigue. Hypothesis 1 is as follows.

*Hypothesis 1a*: *An employee’s family demands increase his or her own fatigue*. *(Pathway A)*
*Hypothesis 1b*: *An employee’s job demands increase his or her own fatigue*. *(Pathway B)*

With regard to the crossover effect of demands on partner’s fatigue, we suggest that this may take the form of an indirect effect through the mutual influence of fatigue—namely, husband’s fatigue influencing wife’s fatigue, and wife’s fatigue influencing husband’s fatigue. This process can be explained by the crossover mechanisms proposed by Westman[6]. Crossover may occur through a direct empathic reaction in the other partner. Persons in a close relationship imagine their partners’ circumstances and how they would feel in their situation, and in doing so, they experience their partners’ feelings. Indeed, a recent study with Japanese couples on the crossover of work engagement (including vigor), showed that the crossover was more pronounced when both partners showed high levels of perspective taking[25]. Additionally, crossover may occur through behaviors towards partners. Stress may lead one partner to engage in negative behavior towards the other partner, thereby increasing distress in the other partner[26]. There is evidence for the mutual influence of burnout[8,24,27], work-family conflict[11,28], distress[29], and positive outcomes[8,15,30]. Consequently, we consider that there is mutual influence of fatigue between dual-earner parents, and, together with Hypothesis 1, that an employee’s job and family demands will increase their partner’s fatigue indirectly through the effect of one’s own fatigue. Hypothesis 2 is as follows.

*Hypothesis 2a*: *An employee’s family demands indirectly increase his or her partner’s fatigue through the effect of mutual influence of fatigue between partners*. *(Pathway A→Pathways C1 and C2)*
*Hypothesis 2b*: *An employee’s job demands indirectly increase his or her partner’s fatigue through the effect of mutual influence of fatigue between partners*. *(Pathway B→Pathways C1 and C2)*

For these crossover effects, two underlying processes are assumed: direct empathic reaction and behaviors toward the partner. Regarding the direct empathic reaction, husbands and wives may differ in their ability to read emotions in their partners. Several studies have confirmed that wives are more accurate than husbands with respect to emotion recognition[31–33]. Thus, wives may potentially be more sensitive to direct empathic crossover. On the other hand, with regard to the crossover process through behaviors towards partners, the results are mixed in terms of gender differences. Consequently, we suggest that the crossover effect of husbands’ fatigue on wives’ fatigue will be stronger than vice versa. Hypothesis 3 is as follows.

*Hypothesis 3*: *The effect of a husband’s fatigue on his wife’s fatigue is stronger than the effect of a wife’s fatigue on her husband’s fatigue*. *(Pathway C1 > Pathway C2)*

In regard to the correlation between an employee’s job demands and his or her partner’s family demands, we anticipate a positive association. This is because high job demands may inhibit the employee from participating in family tasks, which in turn increases his or her partner’s family demands in an attempt to compensate for the decreased effort of the spouse. Previous studies[2,34,35] have confirmed this positive association. Thus, we consider that one’s own job demands will be positively related to his or her partner’s family demands.

*Hypothesis 4*: *An employee’s job demands are positively related to his or her partner’s family demands*. *(Pathway D)*.
*Hypothesis 5*: *An employee’s job demands are positively related to his or her partner’s fatigue through the partner’s family demands*. *(Pathway D→Pathways A and B)*.

## Materials and methods

### Participants and procedure

The present study is a part of the Tokyo Work-Family Interface (TWIN) study, a two-wave cohort study with a one-year time interval. The TWIN study aims to examine the intra-individual and inter-individual processes of well-being in all dual-earner couples with preschool children in the Setagaya Ward in Tokyo, Japan. In the present study, we analyzed the first wave of data collected in 2008. Working partners were approached through the nursery schools. With the help of the Child-raising Assistance Department of Setagaya ward in Tokyo, we sent letters explaining the aims, procedure, and ethical considerations of the study to all directors of nursery schools in this ward. Out of a total of 82 schools, 81 agreed to participate. We distributed two identical questionnaires, one for each partner, through the nursery schools. Respondents returned their questionnaires in closed, pre-stamped envelopes to a researcher at the University of Tokyo. Of the 8,964 questionnaires distributed, 2,992 were returned, resulting in a response rate of 33.4%. The participants in the present study were 2,502 parents (i.e., 1,251 couples) who met the following five criteria: (a) had at least one child six years of age or younger, (b) had a partner (neither widowed nor divorced), (c) lived in a dual-earner household, (d) was employed at least 20 hours per week according to the criteria of Frone [36], and (e) answered all the items in the questionnaire.

The whole procedure followed in the study was reviewed and approved by the Ethics Committee of the Graduate School of Medicine, University of Tokyo.

### Measures

Job demands were operationalized by the quantitative demands of work (work overload). This was measured with four items developed by Furda [37] that refer to quantitative, demanding aspects of the job. These items were validated in a previous study[38]. Sample items are “Do you work under time pressure?” and “How often do you have to work extra hard to finish something?” Items are scored on a five-point scale ranging from “never” to “always.”

Family demands were evaluated by asking employees about the amount (quantitative) family demands they experienced. This was measured by five items developed by Peeters [22] referring to quantitative burdens at home. Sample items are “Do you often have to do things in a hurry at home?” and “Are you busy at home?” Items are scored on a five-point scale ranging from “never” to “always.”

Fatigue was measured with a subscale of the Brief Job Stress Questionnaire (BSJQ) [39]. The scale includes three items that refer to fatigue experienced within the last one month (this period was used only for measuring fatigue). Sample items are “I have felt extremely tired” and “I have felt exhausted.” Items were scored on a four-point scale ranging from “never” to “always.”

Summated scores of the items of each scale were used to create manifest variables for the analysis.

### Data analyses

Data were examined following the recommendations provided by Kenny et al.[40] for evaluating a non-recursive dyadic effect model using structural equation modeling (SEM). In this model, there is covariance between the errors of the husband’s and the wife’s fatigue. The importance of setting this covariance is emphasized in several studies[41,42]. Mplus software (version 7.11)[43] was utilized in the present study. Firstly, we conducted SEM analysis to test the model as displayed in Fig 1. Following the recommendations of Hu and Bentler [44], a good model fit was indicated by scores of .90 or higher on the CFI and scores under .05 for the RMSEA and SRMR parameters. This was followed by an additional analysis to show how much the effects were influenced by possible confounders. We included work-related factors such as working times, shift work (working at night), shorter working time, and commuting time to workplace in the proposed model and allowed to correlate with all other model variables. Secondly, we calculated the magnitude of indirect effects and tested their significance. For this process we utilized the “MODEL INDIRECT” command of Mplus software, which estimates and tests specific indirect effects[45]. Thirdly, to test whether the crossover path from husband to wife is stronger than the effect from wife to husband, we compared the incremental fit of a constrained model in which the crossover parameters were specified to be equivalent to that of the hypothesized model in which the parameters were freely estimated.

## Results

Table 1 shows the demographic characteristics of the study sample for both genders. Husbands were slightly older than wives. In terms of occupational sector, over two thirds of the husbands (70.5%) and more than half of the wives (58.6%) worked for private (vs. public) organizations. More wives (11.2%) worked as civil servants than husbands (8.4%) did, whereas more husbands (12.8%) than wives (10.4%) were self-employed. Regarding job contracts, the vast majority of the husbands (95.6%) and less than three quarters of the wives (70.5%) worked as full-time workers. Regarding work and commuting hours, husbands had significantly longer working hours (9.9 and 7.5, respectively), and slightly longer commuting hours than wives did (1.5 and 1.4, respectively). As for the shorter working hours system, most husbands (97.5%) did not use this system, whereas about one quarter of the wives (25.3%) did (see Table 1).

**Table 1:** Comparison of the demographic characteristics between husbands and wives.

		Husbands				Wives				Statistical test	p value
		n^a^	Mean	SD^b^	%	n	Mean	SD	%		
Age		1227	38.1	5.4		1238	36.3	4.3		t(1215) = 15.20^c^	< .0001
Occupation	Worker for private company	864			70.5%	718			58.6%	χ(3) = 88.263	< .0001
	Civil servant	103			8.4%	137			11.2%		
	Self-employed	157			12.8%	128			10.4%		
	Others (teachers, students etc.)	102			8.3%	254			20.7%		
Job contract	Full-time(≥40 hours/week)	1165			95.6%	863			70.8%	χ(2) = 363.166	< .0001
	Part-time(≤40 hours/week)	14			1.1%	286			23.5%		
	Others	40			3.3%	83			6.8%		
Work (hours/day)		1187	9.9	2.5		1197	7.5	1.8		t(1143) = 26.99^c^	< .0001
Commuting (hours/day)		1133	1.5	0.8		1093	1.4	0.8		t(1016) = 2.73^c^	0.0064
Shorter working hours system	Yes	31			2.5%	316			25.3%	χ(1) = 309.951	< .0001
	No	1220			97.5%	935			74.7%		

^a^ The numbers do not add up to the total number of the participants because of occasional missing data.

^b^ Standard Deviation.

^c^ Paired *t*-test.

Table 2 shows the means, standard deviations, internal consistencies (Cronbach’s alpha), and correlations between the study variables. As can be seen, all variables have acceptable reliabilities with Cronbach’s alpha coefficients of .70 or higher. Fatigue of husband and wife were significantly correlated (r = 0.14, p < .01).

**Table 2:** Means, standard deviations, Cronbach’s alpha, and correlations for the model variables.

	Mean	SD^a^	α	1	2	3	4	5	6
1. Husband's job demands	13.43	4.12	.89	―					
2. Husband's family demands	14.37	4.13	.79	.17**	―				
3. Husband's fatigue	6.72	2.48	.89	.37**	.22**	―			
4. Wife's job demands	12.36	4.25	.89	.07**	.11**	.05**	―		
5. Wife's family demands	19.65	4.12	.81	.04**	.15**	.06**	.23**	―	
6. Wife's fatigue	7.14	2.61	.89	.08**	.14**	.12**	.29**	.29**	―

*p < .05.

**p < .01.

^a^Standard deviation.

**Fig 2:**
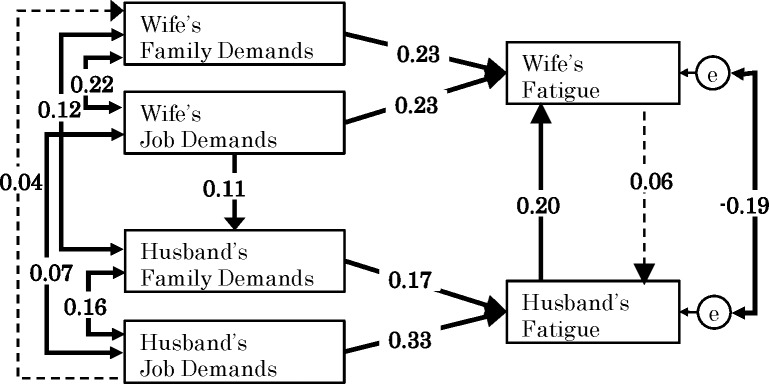
A crossover model of associations between job and family demands and fatigue. Estimates represent standardized coefficients. Significant paths (p <0.05) are shown with solid lines. Non-significant paths are shown with dotted lines.

Fig 2 shows the standardized solution of the hypothesized model. The fit indices for the SEM model indicated a good model fit (χ^2^(2) = 3.011, p = 0.22; RMSEA = 0.02; CFI = 0.998; TLI = 0.987; SRMR = 0.008). Please note that this good fit is mostly due to the model including only observed variables. As for the adjustment of possible confounders, after controlling for confounding variables, the path coefficients remained virtually the same as those in the proposed original model. This indicates that the impact of the control variables on the model variables was weak. Importantly, most control variables did not affect the structural paths in the model significantly (i.e., 53 out of 76 paths were not statistically significant). Therefore, the control variables were removed from the final model in Fig 2.

Husbands’/wives’ job demands and family demands had a positive relationship with their own fatigue. These results provide support to both Hypothesis 1a and 1b.

As for the mutual influence between the husband’s and wife’s fatigue, husband’s fatigue had a positive effect on wife’s fatigue whereas wife’s fatigue did not have a significant effect on husband’s fatigue. Table 3 shows the estimates and p-values of the indirect crossover effect. Both indirect effects of (a) husband’s family demands and (b) husband’s job demands on wife’s fatigue through husband’s fatigue were significant (although the magnitude of the effect was small), whereas both indirect effects of wife’s family and job demands on husband’s fatigue through wife’s fatigue were non-significant. Thus, Hypothesis 2a and 2b were partially supported.

**Table 3:** Indirect pathways from work demands to partner's fatigue through partner's family demands.

		Estimate (95% CI^d^)	p-value
**Effects on husband's fatigue**			
	Wife's job demands → Wife's fatigue→Husband's Fatigue	.014 (-0.018, 0.046)	.394
	Wife's family demands → Wife's fatigue→Husband's fatigue	.014 (-0.019, 0.047)	.396
	Wife's job demands → Husband's family demands →Husband's fatigue	.018 (0.007, 0.028)	.001
**Effects on wife's fatigue**			
	Husband's job demands → Husband's fatigue→ Wife's fatigue	.068 (0.025, 0.111)	.002
	Husband's family demands → Husband's fatigue→ Wife's fatigue	.034 (0.009, 0.058)	.007
	Husband's job demands → Wife's family demands→ Wife's fatigue	.010 (-0.003, 0.020)	.150

^d^ CI = Confidence Interval.

Table 4 shows the model comparison to further test whether the effect of husband’s fatigue on wife’s fatigue is stronger than the effect of wife’s fatigue on husband’s fatigue. We compared the hypothesized model with the model that constrained the parameters of both paths to be equal. The constrained model showed a significantly worse fit compared to the hypothesized model (Delta →^2^(2) = 6.267, p< .05), indicating that the effects were different for husbands and wives. The effect of husband’s fatigue on wife’s fatigue was found to be stronger than the effect of wife’s fatigue on husband’s fatigue. Thus, Hypothesis 3 was supported.

**Table 4:** Fit indices for crossover models.

Model	AIC	χ2	df	RMSEA	CFI	TLI	SRMR	△χ2	△df	p-value
Hypothesized model	39126.5	3.011	2	.020	.998	.989	.008			
Constrained model	39128.7	9.278	4	.032	.987	.971	.016	6.267	2	< .05

Regarding the association between one’s own job demands and one’s partner’s family demands, the effect of wife’s job demands on husband’s family demands was identified as significant, whereas the effect of husband’s job demands on wife’s family demands was not significant (as shown in Fig 2). Consequently, Hypothesis 4 was partially supported.

The indirect effects shown in Table 3 indicate that the effect of wife’s job demands through husband’s family demands on husband’s fatigue was identified as significant (although the magnitude of the effect is small). On the other hand, the indirect effect of husband’s job demands through wife’s family demands on wife’s fatigue was not significant. Accordingly, Hypothesis 5 was partially supported.

## Discussion

The present study investigated the crossover influence of fatigue between parents with a structural equation modeling approach, using the dyad simultaneously. In what follows, we will discuss our findings in detail.

As to our hypothesized positive association of employee’s family and job demands with their own fatigue (Pathway A and B, respectively), our results were supportive. This is in line with our predictions and previous studies[11,18,20–23]. This finding is also consistent with the health-impairment or strain process proposed by the JD-R theory[18,19,46,47]. Our study also showed that the JD-R model can be extended to the area of the home for both partners. This suggests that both demands may independently exacerbate fatigue.

Further results partially supported our hypotheses in which we predicted that an employee’s job/family demands indirectly increase his or her partner’s fatigue through the mutual influence of fatigue between partners (Pathway A → Pathways C1 and C2 and Pathway B → Pathways C1 and C2, respectively): the mutual influence of fatigue between parents did exist as hypothesized, but only for the effect from husband to wife, indicating that the influence was unidirectional. It was shown that if husbands are tired, this will affect wives, but no such phenomenon occurs in the opposite direction. This gender difference was confirmed through further analysis of an incremental Χ^2^ test. Therefore, the hypothesis that the crossover effect of fatigue is stronger from husbands to wives than vice versa (Pathway C1 > Pathway C2) was supported. This is consistent with the theory that women are more accurate in their ability to read emotions than are men[31–33]. When comparing this result with those of previous crossover studies, it is in line with studies indicating unidirectional crossover only from husband to wife[8,15,29], though contrary to the results of studies of exhaustion[8] and distress[29] that indicated unidirectional crossover from wife to husband and bidirectional crossover of burnout[27]. Our result may be the consequence of the context in which the participants lived. Gender inequality is greater in Japan than in many other advanced nations. Regarding the Global Gender Gap Index[48], Japan is ranked 111th out of 144 countries. In addition, gender role differences are likely to be large in Japan. According to the survey of Women’s Activity Promotion[49], up to 46.5% men and 43.2% women in Japan agree with the view that husbands should work outside the home and wives should devote themselves to the household. Social norms with gender roles are internalized by both husbands and wives in Japan. Consequently, wives in Japan may be more affected by family circumstances when compared to wives in Western countries. Japanese wives’ happiness was found to be related to their husbands’ income rather than their own, whereas U.S. wives’ happiness was related to their own income and not their spouses’[50]. As such, in Japan, wives’ fatigue may increase according to their husbands’ fatigue, whereas husbands’ fatigue will not be affected by their wives’ fatigue.

Finally, regarding our hypothesis that there is a positive association between one’s own job demands and one’s partner’s family demands, leading to an indirect effect on partner’s fatigue, only the association between wife’s job demands and husband’s family demands was confirmed to exist. This result opposes that of Bolger’s and Pittman’s study[34,35], which demonstrated the influence of only husband’s job demands on his wife’s circumstances and not vice versa. This gender-specific result is also contrary to the gender-neutral results of Bakker and his colleagues[2], where there were no gender differences regarding the process of job demands affecting home demands. Again, these inconsistent results may be due to differences in the cultural context. As mentioned above, gender inequality is greater in Japan. Therefore, wives bear much of the responsibility for household chores and childcare in Japan[14]. According to the Survey on Time Use and Leisure Activities[51], husbands in Japanese dual-earner parents spent only 39 minutes per day on housework, whereas wives spent as much as 293 minutes per day. Consequently, if wives are unable to handle family tasks for reasons such as work overtime, this will seriously affect the family situation, as family members usually act on the assumption that wives will do most of the tasks. On the contrary, since the usual dedication of husbands to housework is low, family members are not influenced so strongly by husbands’ availability. However, the magnitude of the support that husbands provide to wives may affect the association between wife’s job demands and husband’s family demands. We conducted further analysis adding husbands’ support (measured by wives) as an adjusting variable. The results showed that there was not much difference when compared to the original model, even after adjusting for husbands’ social support.

The findings in this study expand previous research in three ways. Firstly, there is an indirect effect of job and family demands on partner fatigue through one’s own fatigue. Secondly, there is an indirect effect of job demands on partner fatigue through partner family demands. Thirdly, there is a gender difference in these indirect effects. By including both parents’ job and family demands in the analysis, we were able to show the precise mechanism of how dual-earner parents’ job demands affect family circumstances. These findings can be understood as a work-to-family conflict effect. Although we did not directly ask for the effect, we showed work-to-family conflict by highlighting the indirect effect of husbands’ job demands on wives’ fatigue through wives’ family demands.

This study has several limitations. Firstly, it is based on survey data with self-report measures. In addition to self-report bias, common method variance may also have played a role. Therefore, the true associations between variables might be weaker than the relationships observed in this study, although some research indicates that this is not as problematic as once thought[52]. Secondly, we used a cross-sectional research design. The determination of causal relationships requires further testing through longitudinal studies. Thirdly, the response rate of the survey was relatively low. It is possible that the couples who invested long hours in work and family tasks such as child rearing could not afford enough time to respond to the questionnaire. Additionally, persons who are less interested in work-family issues may not have participated in this survey. Thus, the true associations between variables might be weaker than the relationships observed in this study. Future research should aim to increase the interest in work and family issues among potential participants to reduce this selection bias. Fourthly, levels of fatigue at work could depend on the timing of completing the survey. Since we did not indicate this in the questionnaire, it might vary between participants. However, we asked about chronic fatigue in the preceding month, which may not be so strongly influenced by the timing of answering the survey. Finally, this study focused on couples with preschool children, which limits the generalizability of the current findings not only around the globe but also in Japan. Thus, further research is needed to determine whether we can generalize these findings to other areas and other (Western) countries.

### Implications for future practice

This study has several implications. Our findings show that job and family demands of husbands have a positive influence on wives’ fatigue. This result suggests that protecting men’s well-being is beneficial not only for men themselves but also for family functioning. Employers could consider measures such as reduction of long working hours as additional work-family policies. Furthermore, when considering means to reduce employees’ fatigue, gender differences in the mechanism of fatigue need to be taken into account. Women employees are more affected by partners’ states than are men; thus, assessing family circumstances may be particularly effective for women employees. Additionally, interventions such as preventing excessive job demands among husbands or encouraging them to support their wives at home, particularly if these wives also have paid jobs, might also be effective.

## Conclusion

To conclude, this study of Japanese dual-earner parents used a SEM model to demonstrate that job/family demands increase partners’ fatigue through the process of fatigue crossover between partners. Additionally, job demands increase partners’ fatigue through their own family demands. This study also demonstrated that this crossover process is different for both genders.

## References

[1] Meijman TF, Mulder G. Psychological Aspects of Workload In: Drenth PJD, Thierry H, Wolff CJ, editors. Handbook of work and organizational psychology. 2nd ed. Hove, East Sussex: Psychology Press; 1998.

[2] Bakker AB, Demerouti E, Dollard MF. How job demands affect partners’ experience of exhaustion: integrating work-family conflict and crossover theory. J Appl Psychol. 2008;93(4):901–11. 10.1037/0021-9010.93.4.901 18642992

[3] Sluiter JK, de Croon EM, Meijman TF, Frings-Dresen MHW. Need for recovery from work related fatigue and its role in the development and prediction of subjective health complaints. Occup Environ Med. 2003;60:62–70. 10.1136/oem.60.suppl_1.i62 PMC1765724 12782749

[4] Murdock KW, Lovejoy MC, Oddi KB. An Actor-Partner interdependence analysis of associations between affect and parenting behavior among couples. Fam Process. 2014;53(1):120–30. 10.1111/famp.12059 24438316

[5] Bakker AB, Westman M, Hetty van Emmerik IJ. Advancements in crossover theory. J Manag Psychol. 2009;24(3):206–19.

[6] Westman M. Stress and strain crossover. Hum Relations. 2001;54(6):717–51.

[7] Parasuraman S, Greenhaus JH, Granrose CS. Role stressors, social support, and well-being among two-career couples. J Organ Behav. 1992;13(4):339–56.

[8] Demerouti E, Bakker AB, Schaufeli WB. Spillover and crossover of exhaustion and life satisfaction among dual-earner parents. J Vocat Behav. 2005;67(2):266–89.

[9] Jackson SE, Maslach C. After-effects of job-related stress: Families as victims. J Organ Behav. 1982;3(1):63–77.

[10] Jones F. An empirical study of occupational stress transmission in working couples. Hum Relations. 1993;46(7):881–903.

[11] Hammer L, Allen E, Grigsby T. Work–family conflict in dual-earner couples: Within-individual and crossover effects of work and family. J Vocat Behav. 1997;203(50):185–203.

[12] Gorgievski-Duijvesteijin MJ, Giesen CWM, Bakker AB. Financial problems and health complaints among farm couples: Results of a 10-yr follow-up study. J Occup Health Psychol. 2000;5(3):359–73. 10912499 10.1037//1076-8998.5.3.359

[13] Shimazu A, Bakker AB, Demerouti E. How job demands affect an intimate partner: A test of the spillover-crossover model in Japan. J Occup Health. 2009;51(3):239–48. 19390160 10.1539/joh.l8160

[14] Shimazu A, Demerouti E, Bakker AB, Shimada K, Kawakami N. Workaholism and well-being among Japanese dual-earner couples: A spillover-crossover perspective. Soc Sci Med. 2011;73(3):399–409. 10.1016/j.socscimed.2011.05.049 21733607

[15] Westman M, Vinokur AD, Hamilton VL, Roziner I. Crossover of marital dissatisfaction during military downsizing among Russian army officers and their spouses. J Appl Psychol. 2004;89(5):769–79. 10.1037/0021-9010.89.5.769 15506859

[16] Westman M, Vinokur AD. Unraveling the relationship of distress levels within couples: Common stressors, empathic reactions, or crossover via social interaction? Hum Relations. 1998;51(2):137–56.

[17] Morrison DL, Clements R. The effect of one partner’s job characteristics on the other partner’s distress: A serendipitous, but naturalistic, experiment. J Occup Organ Psychol. 1997;70(4):307–24.

[18] Demerouti E, Bakker AB, Nachreiner F, Schaufeli WB. The job demands-resources model of burnout. J Appl Psychol. 2001;86(3):499–512. 11419809

[19] Bakker AB, Demerouti E, Sanz-Vergel AI. Burnout and work engagement: The JD–R Approach. Annu Rev Organ Psychol Organ Behav. 2014;1:389–411.

[20] Maslach C, Schaufeli WB, Leiter MP. Job burnout. Annu Rev Psychol. 2001;52:397–422. 10.1146/annurev.psych.52.1.397 11148311

[21] Bekker MHJ, Croon MA, Bressers B. Childcare involvement, job characteristics, gender and work attitudes as predictors of emotional exhaustion and sickness absence. Work Stress. 2005;19(3):221–37.

[22] Peeters MCW, Montgomery AJ, Bakker AB, Schaufeli WB. Balancing work and home: How job and home demands are related to burnout. Int J Stress Manag. 2005;12(1):43–61.

[23] ten Brummelhuis LL, van der Lippe T, Kluwer ES, Flap H. Positive and negative effects of family involvement on work-related burnout. J Vocat Behav. 2008;73(3):387–96.

[24] Bakker AB. The crossover of burnout and work engagement among working couples. Hum Relations. 2005;58(5):661–89.

[25] Bakker AB, Shimazu A, Demerouti E, Shimada K, Kawakami N. Crossover of work engagement among Japanese couples: Perspective taking by both partners. J Occup Health Psychol. 2011;16(1):112–25. 10.1037/a0021297 21280948

[26] Bakker AB, Demerouti E. The Spillover–Crossover Model In: Grzywacz J, Demerouti E, editors. New frontiers in work and family. Hove, East Sussex: Psychology Press; 2013.

[27] Westman M, Etzion D. Crossover of stress, strain and resources from one spouse to another. J Organ Behav. 1995;16(2):169–81.

[28] Westman M, Da Lia E. The crossover of work-family conflict from one spouse to the other. J Appl Soc Psychol. 2005;35(9):1936–57.

[29] ten Brummelhuis LL, Haar JM, van der Lippe T. Crossover of distress due to work and family demands in dual-earner couples: A dyadic analysis. Work Stress. 2010;24(4):324–41.

[30] Bakker AB, Demerouti E. The crossover of work engagement between working couples. J Manag Psychol. 2009;24(3):220–36.

[31] Hall JA, Matsumoto D. Gender differences in judgments of multiple emotions from facial expressions. Emotion. 2004;4(2):201–6. 10.1037/1528-3542.4.2.201 15222856

[32] Lambrecht L, Kreifelts B, Wildgruber D. Gender differences in emotion recognition: Impact of sensory modality and emotional category. Cogn Emot. 2014;28(3):452–69. 10.1080/02699931.2013.837378 24151963

[33] McClure EB. A meta-analytic review of sex differences in facial expression processing and their development in infants, children, and adolescents. Psychol Bull. 2000;126(3):424–53. 10825784 10.1037/0033-2909.126.3.424

[34] Bolger N, DeLongis A, Kessler RC, Wethington E. The contagion of stress across multiple roles. J Marriage Fam. 1989;51(1):175–83.

[35] Pittman JF, Solheim CA, Blanchard D. Stress as a driver of the allocation of housework. J Marriage Fam. 1996;58(2):456–68.

[36] Frone MR. Work-family conflict and employee psychiatric disorders: The National Comorbidity Survey. J Appl Psychol. 2000;85(6):888–95. 11155895 10.1037/0021-9010.85.6.888

[37] Furda JW. Persoon en welzijn: een toets van het JD-C model [Work, personality, and well-being: A test of the JD-C model]. Unpublished Doctoral Dissertation, Utrecht University (The Netherlands); 1995.

[38] Shimada K, Shimazu A, Bakker AB, Demerouti E, Kawakami N. Work-family spillover among Japanese dual-earner couples: A large community-based study. J Occup Health. 2010;52(6):335–43. 20924151 10.1539/joh.l9130

[39] Shimomitsu T, Yokoyama K, Ono Y, Maruta T, Tanigawa T. Development of a novel brief job stress questionnaire. In: Kato S, editor. Report of the research grant for the prevention of work-related diseases from the Ministry of Labor. Japanese M. Tokyo; 1998. p. 107–115 (in Japanese).

[40] Kenny DA, Kashy DA, Cook WL. Dyadic data analysis New York: Guilford Press; 2006.

[41] Schaubroeck J. Investigating reciprocal causation in organizational behavior research. J Organ Behav. 1990;11(1):17–28.

[42] Wong C-S, Law KS. Testing reciprocal relationships by nonrecursive structural equation models using cross-sectional data. Organ Res methods. 1999;2(1):69–87.

[43] Muthén LK, Muthén BO. (1998–2012). Mplus User’s Guide. 7th ed. Los Angeles, CA: Muthén & Muthen.

[44] Hu L, Bentler PM. Cutoff criteria for fit indexes in covariance structure analysis: Conventional criteria versus new alternatives. Struct Equ Model. 1999;6(1):1–55.

[45] MacKinnon DP. Introduction to statistical mediation analysis Abingdon, Oxfordshire: Routledge; 2008.

[46] Schaufeli WB, Bakker AB. Job demands, job resources, and their relationship with burnout and engagement: A multi-sample study. J Organ Behav. 2004;25(3):293–315.

[47] Demerouti E, Bakker AB. The Job Demands–Resources model: Challenges for future research. SA J Ind Psychol. 2011;37(2):9

[48] World economic forum (2015). The Global Gender Gap Index Results in 2015. Retrieved 2016 Nov 14 from: http://reports.weforum.org/global-gender-gap-report-2015/the-global-gender-gap-index-results-in-2015/

[49] Cabinet office (2014). Survey of Women’s Activity Promotion. Retrieved 2016 Nov 2 from: http://survey.gov-online.go.jp/h26/h26-joseikatsuyaku/gairyaku.pdf (in Japanese)

[50] Lee KS, Ono H. Specialization and happiness in marriage: A U.S.-Japan comparison. Soc Sci Res. 2008;37(4):1216–34. 19227699 10.1016/j.ssresearch.2008.02.005

[51] Ministry of Internal Affairs and Communications (2011). Survey on Time Use and Leisure Activities. Retrieved 2016 Nov 2 from: http://www.e-stat.go.jp/SG1/estat/List.do?bid=000001041121&cycode=0 (in Japanese)

[52] Spector PE. Method variance in organizational research: Truth or urban legend? Organ Res Methods. 2006;9(2):221–32.

